# In Vivo Endomicroscopy of Lung Injury and Repair in ARDS: Potential Added Value to Current Imaging

**DOI:** 10.3390/jcm8081197

**Published:** 2019-08-11

**Authors:** Olivier Lesur, Frédéric Chagnon, Réjean Lebel, Martin Lepage

**Affiliations:** 1 Intensive Care and Pneumology Departments, Faculty of Medicine and Health Sciences, University of Sherbrooke, Sherbrooke, QC J1H 5N4, Canada; 2 Sherbrooke Molecular Imaging Center (CIMS), Faculty of Medicine and Health Sciences, University of Sherbrooke, Sherbrooke, QC J1H 5N4, Canada; 3 Nuclear Medicine and Radiobiology Departments, Faculty of Medicine and Health Sciences, University of Sherbrooke, Sherbrooke, QC J1H 5N4, Canada

**Keywords:** acute respiratory distress syndrome (ARDS), acute lung injury, inflammation, repair, microimaging, endomicroscopy, virtual biopsy, probe-based confocal laser endomicroscopy

## Abstract

Background: Standard clinical imaging of the acute respiratory distress syndrome (ARDS) lung lacks resolution and offers limited possibilities in the exploration of the structure–function relationship, and therefore cannot provide an early and clear discrimination of patients with unexpected diagnosis and unrepair profile. The current gold standard is open lung biopsy (OLB). However, despite being able to reveal precise information about the tissue collected, OLB cannot provide real-time information on treatment response and is accompanied with a complication risk rate up to 25%, making longitudinal monitoring a dangerous endeavor. Intravital probe-based confocal laser endomicroscopy (pCLE) is a developing and innovative high-resolution imaging technology. pCLE offers the possibility to leverage multiple and specific imaging probes to enable multiplex screening of several proteases and pathogenic microorganisms, simultaneously and longitudinally, in the lung. This bedside method will ultimately enable physicians to rapidly, noninvasively, and accurately diagnose degrading lung and/or fibrosis without the need of OLBs. Objectives and Methods: To extend the information provided by standard imaging of the ARDS lung with a bedside, high-resolution, miniaturized pCLE through the detailed molecular imaging of a carefully selected region-of-interest (ROI). To validate and quantify real-time imaging to validate pCLE against OLB. Results: Developments in lung pCLE using fluorescent affinity- or activity-based probes at both preclinical and clinical (first-in-man) stages are ongoing—the results are promising, revealing correlations with OLBs in problematic ARDS. Conclusion: It can be envisaged that safe, high-resolution, noninvasive pCLE with activatable fluorescence probes will provide a “virtual optical biopsy” and will provide decisive information in selected ARDS patients at the bedside.

## 1. Introduction

Our finer understanding of tissue repair and regeneration is not currently capitalized upon by standard imaging methods. This is particularly the case in acute respiratory distress syndrome (ARDS) [1,2,3,4]. Specifically, new lung imaging diagnostic and monitoring technologies to efficiently guide physician decision-making in the intensive care ward are much needed [5,6]. The prerequisites of any such technology are that it should be: easy-to-handle; miniaturized, with portability and bedside affordability, high resolution, tissue penetrance, and innocuity; and it should be able to deliver key-information. This non-exhaustive review paper addresses the current knowledge on lung injury and repair in ARDS. We first discuss lung repair assessment methods in the clinical setting, i.e., standard imaging techniques, with their usefulness and limitations. In this regard, as early as 2008, one of the recommendations in the proceedings of the American Thoracic Society on small animal imaging of the lung was to “utilize imaging modalities to investigate intracellular lung pathophysiology in vivo and in real-time” [7]. More than a decade later, this developing technology area has demonstrated several advantages over standard imaging procedures; however, several challenges still remain before widespread integration into regular medical practice can be achieved.

The pathophysiology of several lung diseases is now known, at least partially, and has identified processes specific to inadequate, dysfunctional, or inappropriate tissue repair (e.g., acute lung injury with ARDS, idiopathic pulmonary fibrosis, and other interstitial lung diseases) [1,8,9,10,11,12]. Specifically, the knowledge gained from experimental studies of acute lung injury in animals (e.g., the bleomycin-, endotoxin-, or hyperoxia-challenged lung models) has provided useful insights into lung repair [13]. Herein, we outline some of the relevant information on the mechanisms of lung repair from preclinical studies and from unpublished human data on ARDS. The objective is to provide the reader with a translational perspective of human conditions and to highlight the potential of pCLE in revealing molecular information on these conditions, acting as an optical “virtual biopsy”.

## 2. Pathophysiological Mechanisms of Lung Repair in ARDS

The mechanisms of lung repair are numerous and are reviewed in detail here. Instead, we summarize the key, pertinent information, and refer the reader to several recent reviews on this topic [1,8,9,10,11,12]. 

The delicate interface between epithelial and endothelial lumen in lung distal airspaces ensures gas exchanges and homeostasis in a unique structure–function relationship. 

The presence of “diffuse alveolar damage” (DAD) and associated injuries are the hallmarks of ARDS [14]. Although variable in severity and diffusion [15], DAD remains a pivotal pathophysiological finding that currently cannot be assessed through a clinical work-up [16].

ARDS is a complex syndrome including different clinical patterns and phenotypes with distinct outcomes. A highly inflammatory sub-phenotype is associated with evidence of continuing lung damage and unrepair with multiple organ dysfunctions and a prolonged need for ventilatory support, linking sustained inflammation to failure in lung tissue restoration [17].

In general, lung injury and repair begin immediately after and in response to a direct or an indirect aggression and can be divided into three phases, which are well ordered and confined to space and time limitations.

An initial triggering event may take diverse forms such as microorganisms (chemical and physical), aggressors, allergens, or circulating immune complexes. In any case, it results in a disruption of the integrity of the alveolar–capillary barrier, with alveolar type I and II epithelial and endothelial cell damage, necrosis, and apoptosis, as well as exposure of the basement membrane [11,18]. 

An inflammatory phase follows this triggering event. It is usually characterized by a protein-rich exudate and by the infiltration of white blood cells such as polymorphonuclear neutrophils (PMNs) and macrophages into the lung tissue and spaces through a leaky blood–gas barrier [19,20,21]. All of the cell types involved (type I and II alveolar epithelium, endothelium, and white blood cells) can produce cytokines and other molecules responsible for the initiation of the repair process and the influx of other cells. The balance of pro-inflammatory and anti-inflammatory mediators will then determine whether the response will be appropriate and self-limited, or inappropriate and enter an out-of-control feedback loop [1,11,18].

A proliferative phase is characterized by epithelial type II cell proliferation and influx of fibroblasts. Type II alveolar cells proliferate, migrate to the damaged area, divide and remodel the underlying basement membrane (partly with the help of fibroblasts), and finally differentiate into type I cells to restore an effective blood–gas barrier. Extracellular matrix deposition and remodeling is a delicate dynamic balance mediated by alveolar epithelial type II cells, myofibroblasts, and macrophages. This is a crucial phase which leads to wound healing and restoration of structure–function that needs to occur inside a time window of 7–12 days [1,8,9,10,11,12,15,18]. If not, a fibrosis/scarring remains in the non-restored area [11,15,22].

Can some of these well-orchestrated dynamic processes be observed in real-time at the cellular and molecular levels? Currently, monitoring these processes in patients requires lung tissue sampling and time-consuming lab processing. A formal diagnosis of continued damage can only be confirmed by open lung biopsy (OLB). This is certainly feasible in well-trained and well-equipped centers with high throughput and tight collaboration between skilled critical care physicians/surgeons and expert pathologists. Although informative in 60%–90% of the cases, especially in steroid-sensitive pathologies, OLBs can have as high as a 25% complication risk rate, making longitudinal monitoring a dangerous endeavor [23,24,25,26,27,28]. Unfortunately, this procedure cannot enable real-time treatment adaptation and personalized care. Although DAD has been an inescapable pathognonomic pathological marker of ARDS for a long time, its presence and extent have been recently challenged [14]. However, DAD can still be a determinant and confirmatory element of the diagnosis. Other useful and discriminant pathological discoveries include infection (e.g., cytomegalovirus or mycobacterial pneumonia), alveolar hemorrhage, cryptogenic organizing pneumonia (COP, formerly bronchiolitis obliterans organizing pneumonia (BOOP)), as well as clues suggesting incomplete repair, such as bundles of collagen fibers in interstitial fibrosis with fibroblastic foci and nests of myofibroblasts [22,23,24,25,26,27,28].

## 3. Evaluation of Lung Repair in ARDS: Clinical Settings

Except for a genuine pathological tissue examination, there are currently no methods for the direct, accurate, and definitive assessment of lung repair (or lack thereof) of ARDS in a clinical setting. After an appropriate diagnosis based on the Berlin definition [29], a supportive therapy is initiated, and the response is assessed by physical examination, standard imaging modalities, and functional tests to monitor the progression of ARDS and the treatment response. The state of lung repair in a specific patient cannot, however, be assessed since none of the above methods enables a clear visualization or quantification of the repair process. These bedside clinical assessments indeed mostly show the initial consequences of function loss, and then return to normal function or further deterioration.

Bedside clinical evaluation allows physicians to determine how lung function is evolving following the initial insult and whether gas exchanges improve or stabilize. Briefly, arterial blood gases with oxygenation index (OI) and/or PaO_2_/FiO_2_ (P/F ratio) are useful to define, stratify, and monitor patients. However, on an individual basis, these clearly lack a fine predictive value regarding the outcome. 

Chest imaging of ARDS using standard imaging techniques is an essential and frequent part of the lung diagnostic work-up. This type of imaging is limited by (1) the challenges associated with moving critically ill/ventilated patients, and (2) a lack of spatial resolution [5,6]. Standard bedside chest X-ray (CXR) can reveal infiltrates, patterns of fibrosis, or relative changes in lung volumes (e.g., atelectasis or pleural effusions). Alveolar or interstitial infiltrates visible by CXR suggest local inflammation, infection, or an increase in membrane permeability. Actually, CXR remains the most useful tool for a dynamic follow-up and/or to discard any critical event at the bedside, despite a lack of specificity regarding the physiological processes at play. Computerized tomography (CT) of the lung provides more detailed anatomical information, thus allowing better characterization of parenchymal abnormalities. For instance, CT can radiologically differentiate the early exudative from the late proliferative and fibrotic phases of acute lung injury (ALI), the former showing ground-glass attenuation and alveolar consolidation and the latter showing traction bronchiectasis and interstitial fibrosis [2,3,4,30]. CT scans allow physicians to identify and differentiate many lung conditions, such as interstitial or consolidated pneumonia, atelectasis, and pulmonary edema. These scans provide better clues than bedside assessment pertaining to the stage and progression of lung repair versus continued damage and unrepair. However, similarly to the methods previously described, they provide little insight into the underlying cellular or molecular processes, and most often require the patient to be transported to the imaging suite with potential related morbidity.

Non-imaging methods of lung investigation include bronchoalveolar lavage (BAL) and OLB. BALs usually provide quantification and differential profiles of recoverable free cells of distal airways, and most importantly, adequate material for microbiological cultures [30]. Inflammatory cytokines can also be quantified and pro- to anti-inflammatory balance evaluated, with regards to lung repair, although the clinical relevance of this information is yet to be determined [31,32]. Unfortunately, although a safe procedure in skilled hands [33], BALs in ARDS patients do not always generate useful microbiological results, nor do they often discriminate between different cell distribution profiles [34]. OLBs and thoracoscopy are used for patients with persisting respiratory failure without a clear underlying cause [23,24,25,26,27,28]. Although rarely performed despite the evidence of diagnostic yield, OLBs enable histological examination of actual lung tissue, thus providing extremely accurate diagnostic information. Microscopic lung architecture and diagnostic elements such as hyaline membranes, white blood cell infiltrates, and collagen distribution can be directly visualized. Specific immunohistochemistry and immunofluorescence can also be performed. Indeed, several groups, as well as metanalyses, have already described the benefits of OLBs in ARDS [23,24,25,26,27,28]. However, the potential morbidity related to the use of an invasive technique on critically ill patients (as mentioned above) and the organizational constraints related to the availability of a specialized lung pathologist limit the clinical use of surgical OLBs. Other disadvantages of OLBs are the questionable representativity and specificity of the selected lung sample—the frequent patchy nature of the insult, the static nature of the specimen, and the potential architectural and molecular alterations related to the fixation process are all further limitations of this approach. 

## 4. Recently Developed Imaging Modalities Clinically Available: Moving Towards the Cellular/Molecular Level?

**CT.** Computed tomography (CT) is undoubtedly the most widely used technique, because of its high spatial/anatomical resolution and its fast image acquisition [35]. Multi-detector CT (MDCT) allows the segmentation of the bronchial tree up to the 5th generation and beyond. These technological advances in CT with increased resolution allow local measurements of the lung (ventilation and volumes) by xenon-enhanced CT (XE-MDCT). The advent of new contrast methods now makes it possible to visualize lung perfusion. Recent studies have shown that coupling a CT scanner with a magnetic resonance imaging (MRI) scanner complements the information obtained [30].

**MRI.** Magnetic resonance imaging (MRI) is also becoming a more widespread technique for lung imaging, especially since the challenges related to low proton density and tissue inhomogeneity have been tackled [30]. Hyperpolarization of gases, notably ^129^Xe, increases their signal by a factor of up to 100,000, thus giving background-free images of these gases [36,37,38]. Movement artefacts of the lung can be attenuated using fast image acquisition techniques, and more complex lung characteristics, can be ascertained by measuring local lung physiology. The advantages of MRI over CT include faster image acquisition and the absence of ionizing radiation. 

**PET.** Positron emission tomography (PET) combined with CT allows physicians to visualize in vivo mechanisms of tissue injury [39]. PET/CT scanners provide 3D images with information on pulmonary structures and on inflammation through enhanced accumulation of ^18^F-fluoro-deoxyglucose (FDG). In addition, type I collagen targeted PET imaging has been recently reported in pulmonary fibrosis and has the potential to assess continued damage in ARDS patients [40]. The main limitation of PET is the modest spatial resolution, leading to partial volume artefacts that can obscure small lesions. The transportation of radioisotopes and radiotracers to different centers is possible but access to a cyclotron is advantageous and the cost is significant. 

**Lung ultrasound (LUS).** Ultrasound imaging is mostly restricted to the visualization of the pleural space because of the high reflection coefficient at the tissue–air interface and at the bone–tissue interface. A new technique, called ultra sound lung comets (ULCs), allows the measurement of the extra vascular water in the lung. This imaging modality is advantageous to monitor ARDS because in this case, the presence of water suggests a thickening of the alveolar wall. In experimental lung injury models reproducing the first stage ARDS, ULCs are capable of quickly detecting extra vascular water and LUS parameters are well correlated to CT imaging findings [41,42,43,44].

The above techniques are useful to obtain structural and physiological information on the lung—usually one at a time. However, PET and MRI are costly and would not allow for daily monitoring of ARDS and its possible rapid evolution in patients. Their spatial resolution and sensitivity are adequate to assess a general overview of the actual state of pulmonary damage and repair. On the other hand, they lack portability and require the movement of the patient to the imaging suite.

## 5. New Precision Imaging Modalities for ARDS: In Vivo Endomicroscopy and Targeted Imaging

Precision medicine is a prerequisite to patient-personalized health approaches. However, personalization depends on acquiring a large amount of data on the patient and the disease—a feat impossible to achieve with classic imaging modalities. PET, MRI, and CT are all mature technologies that unfortunately cannot provide the information needed for physicians treating ARDS patients. Therefore, more effort should now be devoted to in vivo microscopy, which is already a very promising modality that can be further expanded by solving certain engineering challenges. In particular, the potential of pCLE to evaluate pulmonary repair in ARDS is high, because it can provide the molecular or cellular information that physicians need to treat ARDS patients. Validation against OLBs introduces the concept of optical “virtual biopsy” of lung tissues. Specifically, the presence (or the absence) and number of particular cells can reveal whether the lung is repairing or not. The most important characteristics required for this technology include (i) portability (i.e., at the bedside) and miniaturization, (ii) high spatial resolution and penetrance, (iii) innocuity (non-hazardous), and (iv) a fast acquisition time of analytic information.

Portability is essential, as frail ARDS patients should be monitored at the bedside, yet it should be possible to transport a single device between multiple patients. Since most of the time a “through-the-airways” access by flexible bronchoscopy is available (a procedural standard in ICUs), access to the lung is already secured, therefore, the imaging device must fit into a working channel as small as 2 mm. Fitting within a larger bronchoscope allows pCLE devices to reach and explore regions of interest (ROI). The miniaturization of optical devices is progressing rapidly. Several closely related technologies are developing or are already available, such as (1) a Raman spectroscopy endomicroscope prototype, using the molecular vibration and dynamic biochemistry of tissues. (2) Optical coherence tomography (OCT) devices have a micrometer spatial resolution and they provide real-time measurements without the requirement for direct tissue contact. However, this technology does not yet meet requirements (ii) and (iv) for clinical translation [45]; i.e., the limited tissue penetrance and field-of-view still need improvement. As an example of this technology, a clinical-grade commercial endomicroscopic device has recently been commercialized (NvisionVLE^®^ Imaging System, Merit Medical Systems, Inc., South Jordan, UT, USA) [46]. Finally, (3) a two-photon endomicroscope has been recently validated and allows in-depth tissue imaging [47].

Fibered confocal (monophoton) fluorescence microscopes are well ahead of the above-mentioned miniaturized devices, with preclinical and clinically-graded systems that have been available for decades. These have been initially referred to as eCLE (for endoscope-based confocal laser endomicroscopy dedicated to gastrointestinal exploration) and pCLE (for probe-based confocal laser endomicroscopy). However, these technologies still suffer from several limitations (e.g., lack of tissue penetrance, the image acquisition speed is not sufficient, no easy quantitative analyses, variable signal-to-noise ratios, the requirement for tissue contact), but improvements are reported for each of these aspects.

Amongst several homemade and commercial systems, Mauna Kea Technologies has been the first to favor the endobronchial approach. Miniaturized catheters ranging from 1.5 mm to 300 microns and containing a bundle of up to 30,000 optical fiber microprobes guided by a working channel of a regular flexible fiberscope are easy to drive through distal airways to alveoli. Catheters are connected to a fluorescence confocal laser system. The terms “alveoloscopy” and “optical biopsy” have been newly introduced to reflect the information obtained at a cellular and molecular resolution from the deep lung. This system can record video sequences with a relatively fast acquisition rate (up to 12 frames/s) [48]. Spatial resolution and a field-of-view allow the observation of very small structures, much like a regular bench microscope. A clinical-grade apparatus allows lung fluorescence to be measured in the “green” fluorescence spectrum (488 nm excitation wavelength, for fluorescein (FITC) and related fluorophores), while a preclinical dedicated system called Cellvizio™ Dual band is able to image two distinct nonoverlapping fluorescent channels simultaneously (FITC and a “red” near infrared fluorescence spectrum (NIR), 660 nm excitation wavelength).

The FIVE2™ instrument from OptiScan is dedicated to preclinical research. Contrary to the Cellvizio™ system, the FIVE2™ is composed of a single fiber for a user-controlled 3D image stack capability and an adjustable imaging depth allowing the focal plane to be moved from the contact surface to a depth of 400 µm. A single “green” fluorescence spectrum (488 nm excitation wavelength) is available and a transthoracic approach is required. The size and rigidity of the probe (4 mm diameter) preclude access to the airways. The acquisition speed is relatively slow (up to 3.5 frames/s) and recording video sequences is not possible, although with several successive images and the appropriate software, video sequence reconstruction is possible. 

## 6. Advantages, Limitations, and Future Directions for in Vivo Microimaging

**Figure 1:**
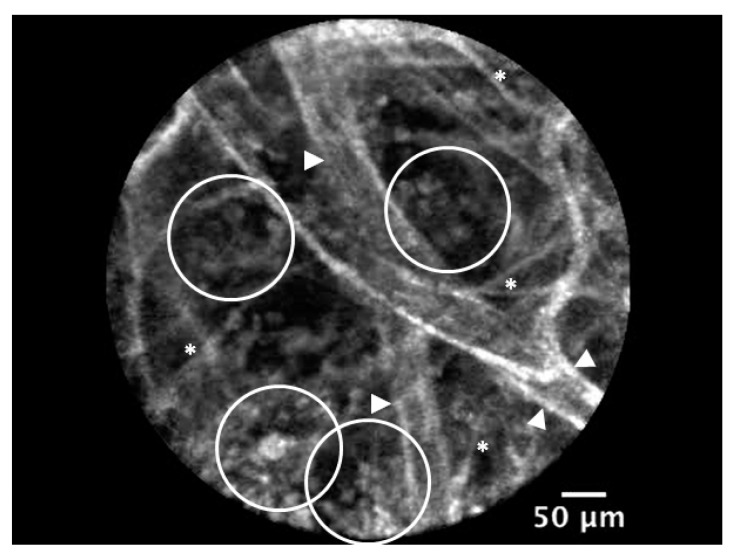
Video capture from a lung endomicroscopy in a patient intubated for a severe community-acquired pneumonia (see Supplementray Materials). Endogenous autofluorescence of distal airspace tissues. The identifiable structures are: numerous infiltrating free cells (presumably polymorphonuclear neutrophils (PMNs) and alveolar macrophages; empty white circles), alveolar micro-vessels (white arrows), septal walls (white asterisks). Cellvizio™ 488 (clinical-grade) (Mauna-Kea Tech.) imaging.

**Figure 2:**
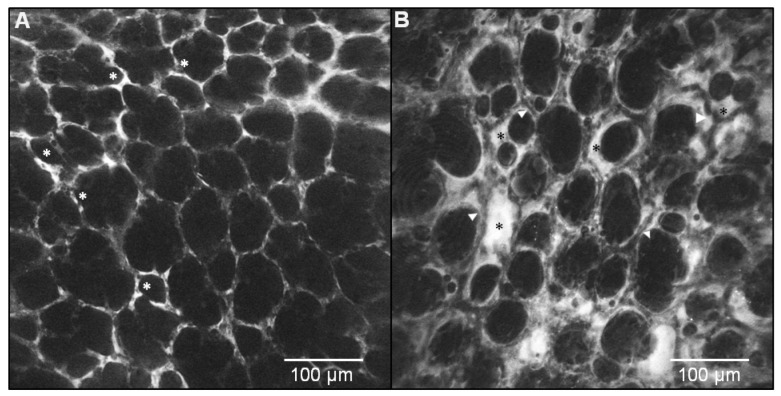
Video captures from a grey-scale video recording of (**A**) a healthy rat lung injected intravenously with a fluorophore (fluorescein)-labeled Dextran (i.e., vascular dye). In view, various filling of alveolar microcirculation (in white) showing capillaries keeping inside the dye (white asterisks). (**B**) A similar view of rat airspaces showing interstitial and alveolar edema after acute respiratory distress syndrome (ARDS) induced by 72 h hyperoxia. Heterogeneous levels of vascular dye leaking with partially/fully flooded airspaces (black asterisks), along with fully/partially empty capillaries (white arrows) [52]. Five 1™ (OptiScan) imaging.

The first approach to in vivo microimaging was to study the autofluorescence of lung and airway tissues without any tracer injection and by working with confocal laser endomicroscopes (CLE) for probe-based CLE (pCLE)—also called fibre-based optical endomicroscopy (OEM) in airways/lung exploration [49]. The endogenous signal is mainly derived from sub-epithelial collagen(s)/elastin (e.g., normal structured network vs. disorganized anarchic dysplasia), from flavines (natural fluorophores), NADH, and FAD containing cells, the number of which being indicative of an inflammatory and/or infectious process [49] (Figure 1). Different structures and patterns of lung autofluorescence can be observed [50], with various profiles depending on the apparatus used, age of tissue, and species. Of note, while traditionally relatively high in adult human lungs, this spontaneous autofluorescence is low to almost undetectable in young rat lung tissues depending on the setting and type of CLE device in use (see below). Additional contrast and information can be gained by the topical or systemic administration of optical fluorescent probes (a few are FDA-approved for human use: e.g., fluorescein, indocyanin green (ICG), methylene blue (MB), cresyl violet) [51,52] or fluorophore-bound macromolecules (e.g., albumin or moderate-to-high molecular weight polysaccharides such as dextran) to visualize and monitor microcirculation and possible real-time extravascular fluid leakage [19,52,53] (Figure 2) which is an essential component of ARDS. Additionally, agglutinins or specific antibodies fragments can specifically enhance the information provided by endomicroscopy imaging [52,54]. Initially limited to preclinical proof-of-concept with antibodies nondedicated to in vivo imaging, this approach was recently shown to refine tissue phenotyping of diseases and to stratify patients for specific therapies [55,56].

**Figure 3:**
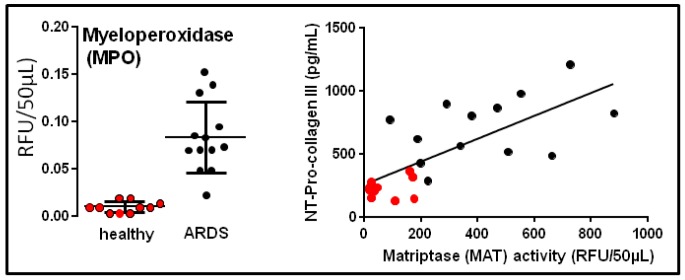
Association between sustained enzymatic protease activities (e.g., myeloperoxidase (MPO), matriptase (MAT)) in bronchoalveolar lavage (BAL) fluids, suggesting unresolved inflammation, and increased levels of N terminal (NT) procollagen III fragments in human ARDS. Individual values obtained in healthy volunteers are in full red circles (*n* = 10), and those of human ARDS are in full black circles (*n* = 13) [67,68,69]. See Supplementary Materials.

**Figure 4:**
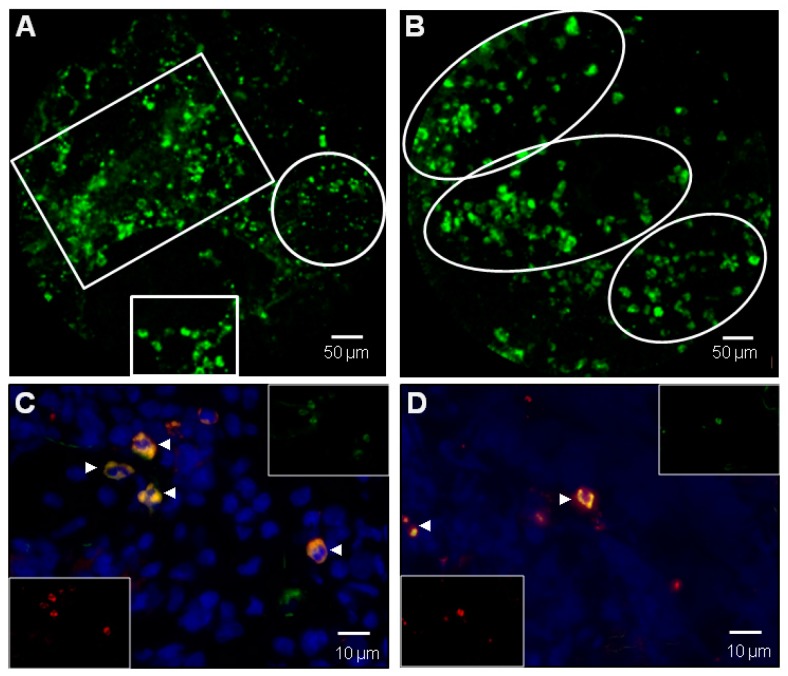
(**A**) Video capture of in vivo endomicroscopy imaging in a rat lung intratracheally instilled with a MPO specific activatable smart probe after 72 h hyperoxia [63]. In view, background fluid edema and airspace structures with numerous dots exhibiting high fluorescence intensity (HFI) concentrated in free inflammatory cells or cluster of cells ranging from 5 to 10 µm (empty white squares) and displaying MPO cellular real-time activation [63]. Nebula of smaller HFI are presumably corresponding to extracellular bio-activation of MPO, especially in Neutrophil (polymorphonuclear neutrophils (PMNs)) extracellular traps (NET)-like structures (empty white circle) [60,61]. (**B**) Another panel of in vivo endomicroscopy imaging showing a low background grouping of distal airspace inflammatory free cells, mainly PMNs, with variable levels of MPO activation (empty white circles). Cellvizio™ Dual Band (Mauna-Kea Tech.) imaging [63]. (**C**) Ex vivo MPO+/elastase + cells in a rat lung after 72 h hyperoxia previously imaged in vivo in panels **(A)** and **(B)**. Coexpression of presumed PMNs is shown in orange (white arrows) and individual stainings are displayed in inserts [63]. (**D**) Similar conditions as in panel (**C)**, showing MPO+/CD68+ cells. Coexpression of presumed alvolar macrophages is shown in orange (white arrows) and individual stainings are displayed in inserts [63].

Another strategy is to use small molecules and peptide-based optical probes (sometimes referred to as “smart probes” [57]) that are specific either by affinity (binding) or activity (enzymatic cleavage). Affinity-based probes can be targeted to receptors, including integrins, some of which are potentially interesting to highlight the vascular system but also to probe remodeling processes (e.g., αvβ3, a commercially product Angiostamp™ is combined with ICG for integrin detection) [58,59]. Activity-based enzymatic probes can be useful to measure “real-time” bioactivity with a specific enzyme. Proteases targeting inflammation (e.g., elastase, myeloperoxidase (Figure 3), cathepsins) and enzymes related to cell death (e.g., caspases) or linked to fibrosis processes (e.g., matriptase, lysyl oxidase) have received attention and some of them have been validated preclinically and tested in human lungs [60,61,62,63,64,65,66,67]. Such probes can potentially inform on the balance between inflammation (sustained or not) and repair vs. fibrosis, which are hallmarks of ARDS. For example, persistent inflammatory hypercellularity and cytokine contents, as well as the presence of high levels of procollagen III or matriptase in BAL fluids (Figure 4) are all indicative of repair abortion and fibrosis in ARDS [67,68].

In addition, recent progress has been made to target the bacteria responsible for hospital-acquired lung infection. Bacterial pneumonia was already recognized as a cause of as much as two-thirds of ARDS in large trials [70]. Under mechanical ventilation, a significant proportion of ARDS patients are susceptible to nosocomial pneumonia, mostly attributed to Gram negative bacteria [70]. Very recently, a topically administered fluorescent smart probe targeting lipid A (a distinctive phospho-glycolipid in the innermost of the three regions of the lipopolysaccharide wall in Gram negative bacteria) has been successfully tested as a diagnostic tool to detect pneumonia in mechanically ventilated patients [70,71,72].

Endomicroscopy in vivo imaging has major advantages in studying lung inflammation and repair over traditional imaging techniques. It is the sole imaging modality able to visualize microscopic lung architecture without the need for collecting tissue, as in OLB. With the use of in vivo probes, it has the potential of providing at least as much information as a biopsy specimen, with the added benefit of repeatedly visualizing actual live tissues. In vivo imaging eliminates confounding factors related to laboratory time-consuming in vitro processing. 

Before in vivo “live” imaging can be commonly used at the bedside, the current limitations and hurdles must be overcome. Images of lung obtained by in vivo microscopes, with or without fluorescent probes, have thus far rarely been seen. Experience at interpreting the images must be solidly acquired before interpretation guidelines and diagnostic criteria as well established as those of conventional microscopy may be published. The use of in vivo fluorescent smart probes is still recent. Much research is needed before an extensive and well-studied panel is characterized, tested, validated, and FDA approved. On this note, topical administration of very low probe concentrations should facilitate approval. Other limitations of in vivo imaging are mechanical. The constant movement of live breathing lungs or beating hearts can render difficult a stabilization of the imaging device in targeted tissue segments. The wedge position of the optical probe through distal airways can at least partially solve this issue. An ideal site of the damaged lung must be correctly identified before the topical injection of the probe. Once an ROI is selected, the same site must be found again to monitor any change that has occurred as a function of the treatment. This is still a significant challenge if the same microscopic area must be found. Very recent technological advancements in pCLE now offer multi-channel and multi-color imaging which allow more precise and extensive real-time mapping and assessment of the tissue condition and repair processes [62,63,64,65,66].

**Figure 5:**
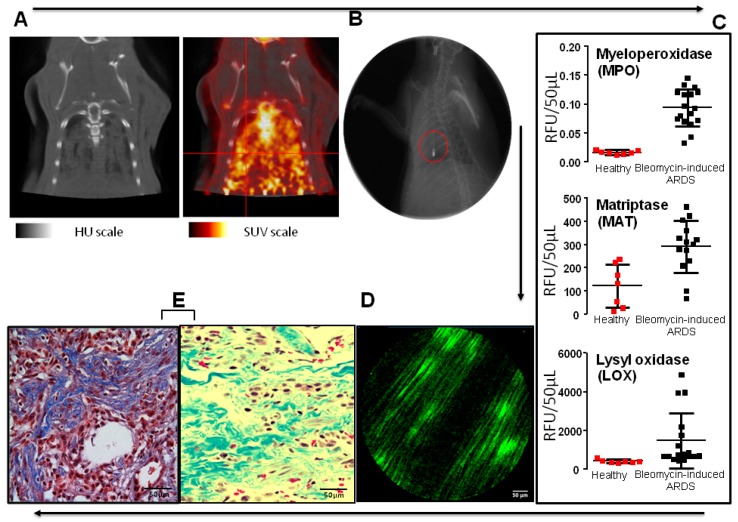
Proposed multimodality testing/imaging sequence in ARDS with unconventional timescale for recovery. Example of a preclinical approach with an experimental model of bleomycin lung in rat. (**A**) Acute lung injury with bilateral infiltrates is documented by PET–CT (respective scales: PET: 0 to 1.84 standardized uptake value (SUV); CT: −1000 to +2000 Hounsfield Units (HU)). Diffuse and enhanced uptake of fluoro-deoxyglucose (FDG) is documented allowing for the selection of a region-of-interest (ROI) for further investigations. (**B**) Endoscopy-guided exploration of a selected ROI (confirmed by X-ray fluoroscopy) is performed and a BAL is carried out. (**C**) Several enzymatic activities measured in BAL fluids reveal the persistence of both inflammation (myeloperoxidase (MPO), matriptase (MAT)) [63] and profibrotic (MAT, lysyl oxidase (LOX)) environments suggesting permanent lesions to lung tissues (*n* = 7 for healthy animals, *n* = 17 for bleomycin-challenged lungs with induced ARDS. (**D**) Endomicroscopy (pCLE) after smart probe systemic injection demonstrating prominence of bundles of type I collagen [74], Cellvizio™ Dual Band (Mauna-Kea Tech.) imaging in a green channel. (**E**) Optical histochemistry of the above **D** tissues from an open lung biopsy (OLB) (Masson-Trichrome staining) confirms the presence of collagen bundles and clusters of fibroblasts in distal airspaces. Panel (**E**) is foreseen to become optional in the near future with the development and validation of the “virtual optical biopsy” concept.

**Figure 6:**
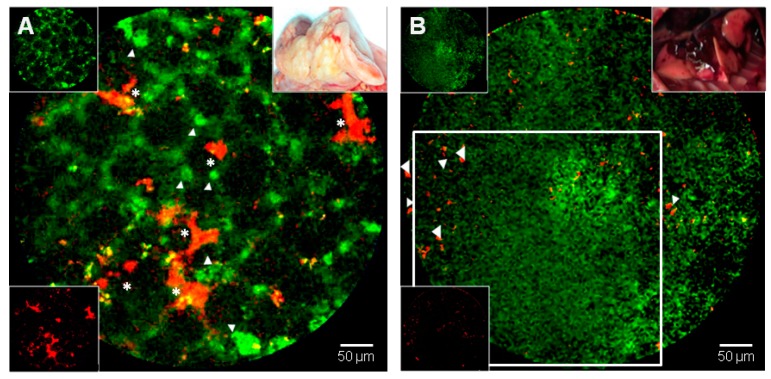
Two video captures exemplifying a dual-band (duplex) preclinical in vivo imaging of lung airspaces in different conditions (see Supplementary Materials). (**A**) Three-weeks after radiation-induced lung injury [75,76,77,78,79,80]. The rat was intravenously injected with fluorescein (FITC)-Dextran (green) and 1,1′-Dioctadecyl-3,3,3′,3′ -Tetramethylindodicarbocyanine-DiD-labeled mesenchymal stromal cells (MSC, in red) seven days before imaging. Green fluorescent dye highlights acinar structures or concentrate in presumed phagocytic cells (white arrows), as well as several areas of grafted red MSC are detectable (white asterisks). (**B**) Two weeks after intratracheal instillation of bleomycin. The rat was intravenously injected with a matriptase (MAT) smart probe and TO-PRO-3, a carbocyanine monomer nucleic acid stain which can access a permeabilized nuclear membrane only in apoptotic cells. A hepatized lung airspace region is shown with loss of traditional acini and septation framework; diffuse MAT capitation/activation (green, open white square) and essentially sparse and peripheral cell nuclear visioning (red, white arrows). Overall, this area would have been defined as a “ground glass opacity” or a “consolidation at a radiological level, or as a ”diffuse alveolar damage” (DAD) at the histopathological level, and is further characterized by a diffuse MAT activation (both representative of inflammation and linked to damaged foci of fibrogenesis with surrounding cell apoptosis at an intra-vital molecular level) [69]. Cellvizio™ Dual Band (Mauna Kea Tech.). A macroscopic appearance of (**A**) and (**B**) in vivo lungs are displayed as both upper right inserts, and individual labeling are displayed in left-side inserts.

In the process of developing new treatment approaches in ARDS, a better understanding of lung repair is a logical step. Current imaging technologies are unable to adequately assess lung repair. There is a huge gap in bedside assessment that may be filled with in vivo microimaging. Patients with a clear early diagnosis of ARDS and a reliable etiology may benefit from in vivo microimaging as a prognostic indicator and as a guide in the timing of treatment initiation and modulation. For instance, the use of corticosteroids in ARDS has been a subject of debate, with large studies showing no clear benefit [73]. Corticosteroids are, however, still useful in subsets of patients and in vivo microimaging may help in identifying them [23]. Patients with an unclear early diagnosis and etiology may also benefit from in vivo microimaging to better discover the causative element(s) and to further identify the parameters of sustained inflammation and sustained tissue damage that may help physicians in the decision-making process. A combination of standard techniques such as CT (and PET) with in vivo microscopy may be useful in this regard. In addition, multiplexed (multiple imaging wavelengths) in situ optical imaging is mandatory to establish a credible and efficient virtual biopsy at the bedside (see the preclinical examples in Figure 5 for an algorithmic multimodality testing/imaging sequence in experimental ARDS, and in Figure 6 for a duplexed lung microimaging, including detection of mesenchymal stem cells (MSC) lung engraftment and visualization of MAT activation and nuclear apoptosis).

The study of lung repair could potentially be of considerable benefit in aiding patients stricken with acute lung diseases such as ARDS. It is of interest for these patients that this research involving in vivo microimaging of the lung be pursued and accelerated.

## Supplementary Materials

The following are available online at https://www.mdpi.com/2077-0383/8/8/1197/s1, File S1: Supplementary Material and Methods.
