# Supplementary material for: In Vivo Endomicroscopy of Lung Injury and Repair in ARDS: Potential Added Value to Current Imaging
Source: J Clin Med. 2019 Aug 11;8(8):1197. doi: 10.3390/jcm8081197 (PMC6723156; doi:10.3390/jcm8081197)
Supplement: Supplementary file 1 [file jcm-08-01197-s001.pdf]

SUPPLEMENTARY MATERIAL and METHODS

Preclinical experimental procedures

Experimental models of ARDS

Pathogen-free adult male Sprague-Dawley rats (10-12 weeks old, 250-350g, Charles Rivers, Can) were used to perform the experimental ARDS which provide the data presented in figures 2 and 4-to 6. All rats received care in compliance with the Guide for the Care and Use of Experimental Animals from the Canadian Council of Animal Care (CCAC; 1993, 2nd ed.). Protocols were approved by the Université de Sherbrooke Ethics Committee for Animal Care and Experimentation (for the Bleomycin and hyperoxia models) and the Animal Care Committee at the Research Institute of the McGill University Health Centre (for the two-to-three weeks post radiation-induced pulmonary fibrosis –RIPF- model)(#038-17BR). 72hrs hyperoxia exposure, Bleomycin 5U/kg intratracheal (i-t) instillation; and RIPF were performed as previously described [1,2]. Endomicroscopy imaging; BALs as well as *ex vivo* lung optical and fluorescence staining were carried out as reported earlier and under established and approved procedures [1].

Positron Emission tomography (PET) and CT scan

Acquisitions. A randomly selected rat challenged 2-weeks ago by Bleomycin i-t infusion (as above) was injected with 18 MBq ^{18}F-FDG in 0.4 ml 0.9% NaCl via the caudal vein over 30 seconds. Thirty minutes later, the animals were positioned into a dedicated LabPet8 PET scanner (Gamma Medica-IDEAS Inc., Sherbrooke, Quebec, Canada), and were scanned for 30 minutes in list-mode with respiratory gating. Then, the rats were scanned by CT (Triumph X-O small animal CT scanner), which is mounted with a 2368 x 2240 CsI flat panel x-ray detector (50 µm pixel pitch in both u and v dimensions), using a 2x2 binning scheme. Data were acquired over 360° using 512 projections (86 mm field-of-view), and the source was operated at a peak voltage of 60 kVp at 240 µA. Air and water phantoms were scanned for each imaging session to allow normalization in Hounsfield Units.

Reconstructions. For PET, a single image was reconstructed with a 3D Maximum Likelihood Expectation Maximization (MLEM) algorithm [3], 20 iterations with resolution of 0.5 x 0.5 x 0.59 mm^3. For CT, the reconstruction was performed as described by Thibaudeau et al. [4] with an isotropic 0.169 mm resolution, 5 iterations, ordered subset acceleration of 32, and tracing oversampling of 5.

X-ray fluoroscopy.

X-ray fluoroscopy guidance (C-Arm, BV Pulsera, Philips Medical Systems, USA) was used in a Bleomycin rat model to pinpoint the positioning of a dedicated Proflex S-1500 experimental probe during fluorescence endomicroscopy imaging and to map it back to its anatomical location in the corresponding CT image [2].

Bronchoalveolar lavage (BAL) and determination of enzymatic activities and collagen propeptide contents in BAL Fluids

All enzymatic activities were performed on 50µL of rat BAL fluids. BALs were carried out immediately after animal sacrifice, under general anaesthesia and at the endpoint of the selected experimental ARDS procedure (*i.e.* 2-to-3 weeks after Bleomycin I/T instillation). Lysyl oxidase (LOX) activity was quantified according to a commercially available fluorometric assay kit (Abcam, Cambridge, MA, USA), myeloperoxidase (MPO) activity, as described previously [1], and matriptase (MAT) activity according to a commercially available fluorometric assay kit (AnaSpec, Fremont, CA, USA).

In vivo fluorescence pCLE imaging

Detection and visualization of lung fibrosis;.distal airspace edema; active inflammation; and mesenchymal stem cell (MSC) grafting, were performed in rat ARDS models as

detailed previously [1-2, 5-6]. The collagen probe used was slightly modified from Désogère et al [7] as follows: Ac-Lys(Ac)-Trp-His-[*Cys-Thr-Thr-K(FITC)-Phe-Pro-His-His-Tyr-Cys]-Leu-Tyr-Bip-Amide, with Gd-DTPA replaced by fluorescein isothiocyanate (FITC). 3.5µM of collagen probe was injected intravenously before the imaging procedure, as detailed [2].The design of the matriptase (MAT) probe used for *in vivo* endomicroscopy was based upon a probe synthesized and validated by Leduc et al. [8]. The *o*-aminobenzoyl was replaced by a fluorescein isothiocyanate (FITC). The matriptase probe design is as follows: FITC-RQRRVVGGK(Dabcyl)-amide. 50µM of MAT probe and 50 µM of TO-PRO-3™ (Life technologies, Burlington, ON, Canada) were intravenously injected, and image recording was done 5-to-15 minutes afterward. MSC were prepared, administered and visualized as reported [6,9].

Ex vivo fluorescence microscopy of lung tissues.

Ex vivo fluorescence microscopy of MPO smartprobe was performed on frozen lung tissues previously explored by endomicroscopy, as described before [1]. Additional phenotype of MPO+ cells was obtained using antibodies against neutrophil elastase and CD68, completed by DAPI nuclear counterstaining, as detailed previously [1].

Human procedures

Bronchoscopy/ endomicroscopy and bronchoalveolar lavages

Autofluorescence imaging was performed during a regular fiberoptic exploration with clinical indication in an intubated-ventilated patient exhibiting a severe community-acquired pneumonia at the ICU ward. Approval for using a clinical-grade sterilized Proflex S-1500 probe from Mauna-Kea Tech. through the working channel of the bronchoscope device was obtained to a legal next of kin and consolidated by the patient own-agreement after recovery.

Bronchoalveolar lavages (BALs) were performed with an official informed consent (Ethics committee project identification code: #2013-476) in accordance with the Declaration of Helsinki. A legal next of kin gave an informed consent for inclusion, and a supplementary patient approval was further secured –when possible- after wake-up, all patients being under deep sedation and intubated-ventilated during the BAL procedure. Healthy volunteers gave an informed consent before the BAL procedure. Following selection of a region-of-interest (ROI) by CT scanning of the lung, and preliminary general overview of the airways, the tip of the bronchoscope was wedged and 5 x 20mL aliquots of normal saline at room temperature were instilled and recovered by gentle suction, as described previously [10].

BAL Fluid measurements

MPO and MAT activities were quantified in BAL fluids as above. N-Terminal Procollagen III propeptide contents were additionally measured using a commercially available human specific ELISA (Novus Biologicals, BioTechne Canada).

References

[1] Chagnon, F.; Bourgouin, A.; Lebel, R.; Bonin, MA.; Marsault, E.; Lepage, M.; Lesur, O. Smart imaging of acute lung injury: exploration of myeloperoxidase activity using in vivo endoscopic confocal fluorescence microscopy. *Am J Physiol Lung Cell Mol Physiol* **2015**, 309(6), L543-51.

[2] Perez, JR.; Ybarra, N.; Chagnon, F.; Serban, M.; Pare, G.; Lesur, O.; Seuntjens, J.; Naqa, IE. Image-Guided Fluorescence Endomicroscopy: From Macro- to Micro-Imaging of Radiation-Induced Pulmonary Fibrosis. *Sci Rep*. **2017**, 7(1), 17829.

[3] Paquette, M.; Vilera-Perez, LG.; Beaudoin, S.; Ekindi-Ndongo, N.; Boudreaut, PL.; Bonin, MA.; Battista, MC.; Bentourkia, M.; Lopez, AF.; Lecomte, R.; Marsault, E.; Guérin, B.; Sabbagh, R.; Leyton, JV. Targeting IL-5Rα with antibody-conjugates reveals a strategy for imaging and therapy for invasive bladder cancer. *Oncoimmunology*. **2017**, 6(10), e1331195.

[4] Thibaudeau, C.; Leroux, JD.; Fontaine, R.; Lecomte, R. Fully 3D iterative CT reconstruction using polar coordinates. *Med Phys*. **2013**, 40(11), 111904.

[5] Chagnon, F.; Fournier, C.; Charette, PG.; Moleski, L.; Payet, MD.; Dobbs, LG.; Lesur, O. In vivo intravital endoscopic confocal fluorescence microscopy of normal and acutely injured rat lungs. *Lab Invest*. **2010**, 90(6), 824-34.

[6] Perez, JR.; Ybarra, N.; Chagnon, F.; Serban, M.; Lee, S.; Seuntjens, J.; Lesur, O.; El Naqa, I. Tracking of Mesenchymal Stem Cells with Fluorescence Endomicroscopy Imaging in Radiotherapy-Induced Lung Injury. *Sci Rep*. **2017**, 7, 40748.

[7] Désogère P, Tapias LF, Hariri LP, Rotile NJ, Rietz TA, Probst CK, Blasi F, Day H, Mino-Kenudson M, Weinreb P, Violette SM, Fuchs BC, Tager AM, Lanuti M, Caravan P. Type I collagen-targeted PET probe for pulmonary fibrosis detection and staging in preclinical models. *Sci Transl Med.* **2017,** 9, 384.

[8] Béliveau, F.; Désilets, A.; Leduc, R. Probing the substrate specificities of matriptase, matriptase-2, hepsin and DESC1 with internally quenched fluorescent peptides. *FEBS J*. **2009,** 276(8), 2213-26.

[9] Perez JR, Ybarra N, Chagnon F, Lesur O, Seuntjens J, Naqa IE. Fluorescence Endomicroscopy Imaging of Mesenchymal Stem Cells in the Rat Lung. *Curr Protoc Stem Cell Biol*. **2018**, 45(1), e52.

[10] Lesur, O.; Kokis, A.; Hermans, C.; Fülöp, T.; Bernard, A.; Lane, D. Interleukin-2 involvement in early acute respiratory distress syndrome: relationship with polymorphonuclear neutrophil apoptosis and patient survival. *Crit Care Med* **2000**, 12, 3814-22.
